# Exosome in HBV infection: current concepts and future perspectives

**DOI:** 10.3389/fcimb.2025.1547525

**Published:** 2025-07-23

**Authors:** XueLan Yuan, ChunXia Huang, Yan Ran

**Affiliations:** ^1^ Health Management Center, Fengdu General Hospital, Fengdu County, Chongqing, China; ^2^ Department of Gastroenterology, Fengdu General Hospital, Fengdu County, Chongqing, China; ^3^ Department of Infectious Diseases, Fengdu General Hospital, Fengdu County, Chongqing, China

**Keywords:** HBV, exosome, immune response, virus infection, therapy

## Abstract

Exosomes are nanoparticles delimited by a lipid bilayer that are secreted by a wide variety of cells. They play a significant role in the pathophysiological processes associated with HBV infection, which poses huge burdens for public health. Emerging evidence has been demonstrated that exosomes are extensively associated with the replication and transmission processes of HBV. In this review, we outline the process of exosome production, composition and function. Moreover, we elaborate on the essential role of exosomes in the pathology processes of HBV infection. Exosomes could serve as essential biomarkers for early detection of HBV infection and HBV-related diseases. Finally, we outline the therapeutic potential of exosomes in anti-HBV therapy, which may provide novel insights into the prevention and treatment of HBV.

## 1 Introduction

Hepatitis B virus (HBV) is a specific small hepatotropic DNA virus responsible for hepatitis B, currently recognized as the most prevalent serious liver infection worldwide (Shih et al., 2018; Zheng et al., 2024). Chronic hepatitis B virus infections impact approximately 250 million individuals worldwide (Lai-Hung Wong and Lemoine, 2024). As a hepatotropic virus, HBV often progresses to cirrhosis and hepatocellular carcinoma (HCC) in patients with high HBV DNA (Starnawski et al., 2025). Globally, 60% to 80% of HCC cases are attributed to HBV infection (Starnawski et al., 2025). Additionally, approximately 70% of cirrhosis cases are caused by HBV (Zhang S. et al., 2025). Despite the development of effective preventive vaccines and oral antiviral medications, chronic HBV infection continues to be a significant underlying or contributing factor to a considerable health burden (Bixler et al., 2019).

Extracellular vesicles (EVs) are nanoscale membrane vesicles released actively by cells (Colombo et al., 2014). Based on their biogenesis, size, and biophysical properties, they can be further classified into subtypes such as exosomes and microvesicles (Wang et al., 2025). Initially regarded merely as cellular debris, EVs are increasingly recognized as important mediators of intercellular communication and significant carriers of circulating biomarkers for disease diagnosis and prognosis (Lorite et al., 2024; Zhang L. et al., 2025). Exosomes are derived from endosomal origins and typically range in size from approximately 40 to 160 nm in diameter, with an average size of around 100 nm (Krylova and Feng, 2023). All exosomes carry a variety of active substances, including proteins, lipids, and nucleic acids, and are crucial for intercellular communication when released into the extracellular environment (Colombo et al., 2014; Welsh et al., 2024). They function as cargo vehicles in various biological fluids, such as serum, plasma, urine, saliva, breast milk, pleural effusions, bronchoalveolar lavage fluid, epididymal fluid, and amniotic fluid (Kalluri and LeBleu, 2020). The current characterization of the biological activities of exosomes primarily relies on non-physiological readouts generated from tissue cultures, which may be amplified, as well as various EVs isolation methods that require further refinement (Zhang et al., 2023). Nevertheless, the role of exosomes in cellular function appears to extend beyond mere protein recycling and varies according to the cellular origin, metabolic status, and environment (Liang et al., 2021). Molecules contained within exosomes, such as signaling proteins and RNA, can be internalized by recipient cells, thereby influencing their physiological state and function (Kim et al., 2024). This enables effective information transfer between the originating and receiving cells (Kim et al., 2024). Exosomes play a crucial role in mediating communication among immune cells and regulating immune responses (Li et al., 2025). For instance, exosomes released by tumor cells can suppress immune system activity, aiding tumor cells in evading immune surveillance (Liu et al., 2025). Additionally, exosomes are important in the processes of tissue repair and regeneration; they facilitate the proliferation and migration of adjacent cells by delivering growth factors and cytokines (Khalilzad et al., 2025). The investigation of exosomes and the mechanisms that regulate their cellular bioactivities and functions encompasses a wide range of processes (Hu et al., 2023). Exosome research is driven by its potential to function as biomarkers for treating numerous human diseases including: cardiovascular dysfunction, neurodegenerative disorders, and tumors (Barile and Vassalli, 2017). Recently, emerging evidence has revealed the essential role of exosomes in HBV infection, which is attributed to their structural and functional similarities (Liu et al., 2023; Peng et al., 2023). Given that exosomes are found in all biological fluids and possess the potential for multicomponent analyses, their application as liquid biopsies are particularly promising (Li et al., 2024).

In this review, we provide an overview of our current knowledge regarding the pathogenesis of HBV, with an emphasis on the role of exosomes in disease development and their potentially application into anti-HBV therapy. We also underscore the future prospects for clinical translation in this field.

## 2 The source and function of exosomes

EVs from endosomal origin were initially discovered in sheep reticulocytes in 1983 and subsequently named “exosomes” in 1987 (Pan and Johnstone, 1983; Johnstone et al., 1987). Research has demonstrated that exosomes are secreted *in vitro* by diverse eukaryotic cell types and can be found in bodily fluids, including blood, urine, bile, synovial fluid, breast milk, and semen (Abels and Breakefield, 2016). The protein and nucleic acid components within EVs are shielded from degradation by the vesicular membrane structure, rendering them markedly more stable than their free counterparts in human body fluids (Gupta et al., 2021). Exosomes originate from multivesicular bodies (MVBs), which are formed by the inward budding of the plasma membrane (Arumugam and Kaur, 2017). These early endosomes subsequently mature into late endosomes and MVBs that contain intraluminal vesicles (ILVs) (Han et al., 2022). After the formation of ILVs, some MVBs fuse with the cell membrane, releasing exosomes into the extracellular space, while others are degraded by lysosomes (Liu et al., 2021). The secretion of exosomes is mediated by the endosomal sorting complex required for transport (ESCRT), a family of proteins composed of four distinct complexes: ESCRT-0, ESCRT-I, ESCRT-II, and ESCRT-III (La Torre et al., 2024). Different ESCRT complexes display distinct functions in biological activities. ESCRT-0 mediates cargo aggregation through a ubiquitination-dependent pathway, while ESCRT-I and ESCRT-II initiate membrane budding. ESCRT-III is responsible for vesicle scission, and accessory proteins, such as VPS4 ATPase, facilitate the disassembly and recycling of the ESCRT complex (Aseervatham, 2023; Rivera-Cuevas and Carruthers, 2023).

**Figure 1:**
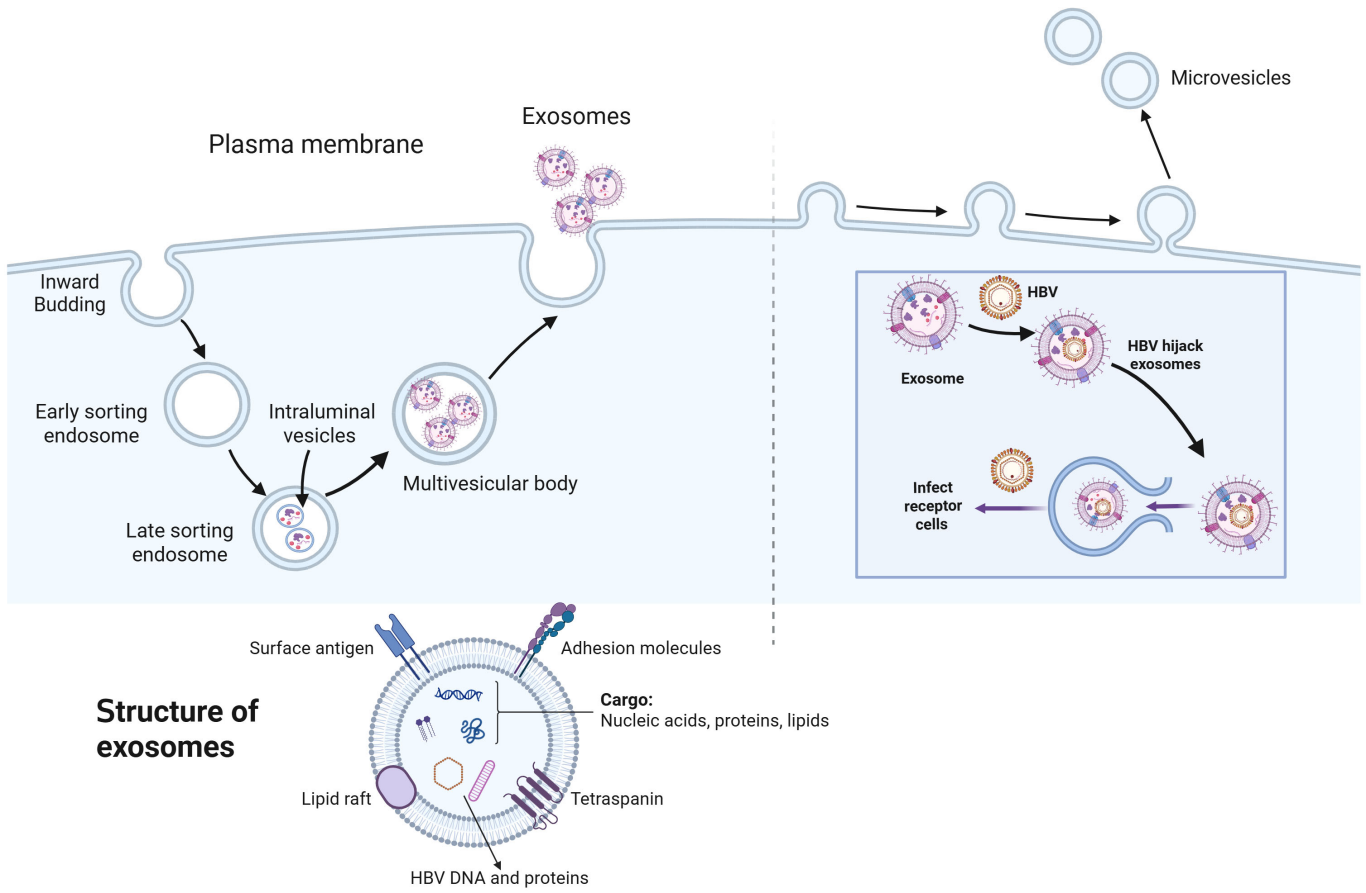
The biogenesis of exosomes and the essential role of exosomes in HBV infection. Exosome biogenesis is a multistep process beginning with the inward budding of the plasma membrane, which engulfs extracellular components to generate early endosomes. These early endosomes subsequently merge and mature into early sorting endosomes, which undergo a series of sorting and trafficking events, eventually differentiating into late sorting endosomes (LSEs). Within LSEs, further invagination of the endosomal membrane forms intraluminal vesicles (ILVs), resulting in the development of multivesicular bodies (MVBs). When MVBs fuse with the plasma membrane, ILVs are released as exosomes into the extracellular space via exocytosis. The process of microvesicle formation begins with the generation of outward protrusions at specific locations on the membrane, followed by fission and subsequent release of the vesicle into the extracellular space. Exosomes are small extracellular vesicles ranging from 30 to 150 nanometers in diameter, characterized by a lipid bilayer membrane composed of phospholipids and cholesterol. Their surface is covered with proteins such as tetraspanins and major histocompatibility complex molecules, which facilitate interactions with recipient cells. Internally, exosomes contain a diverse array of biomolecules, including proteins, various types of RNAs, and lipids. Moreover, they also include the hepatitis B virus (HBV) DNA and HB-related proteins upon HBV infection. Additionally, hepatitis B virus (HBV) exploits exosomes as vehicles for its release, transport, and interaction with target cells. Upon infecting host cells, HBV produces viral proteins and nucleic acids, which are subsequently packaged into exosomes, facilitating infecting receptor cells.

These EVs offer significant potential as biomarkers for liquid biopsies and therapeutic applications, functioning as an innovative mechanism for intercellular communication and molecular transport (Čeri et al., 2024). These biovesicles are secreted by nearly all cell types and transport bioactive proteins, lipids, and nucleic acids (including noncoding RNAs) to target cells at distant locations (Palma et al., 2023; Dai Y. et al., 2024). Exosomes have emerged as potential diagnostic and prognostic biomarkers for various diseases, including cardiovascular conditions, cancers and neurodegenerative disorders (Chang et al., 2024; Yang et al., 2024). Recently, emerging evidence has demonstrated that exosomes play critical roles in viral infection (Chavda et al., 2024). During these processes, exosomes are integral to host immunity, as they contribute to the activation of antiviral responses and mediate the transfer of antiviral factors between adjacent cells (Shi et al., 2024). Exosomes influence the viral infection process in two ways: by modulating their cargo and by regulating the immune status of the host, which provides novel insights into the antiviral therapy (Shi et al., 2024) (**Figure 1**).

## 3 Exosomes in the pathology processes of HBV infection

Many viruses rely on intracellular trafficking to complete replication, initially interacting with the cell surface and following pathways to endosomes. The assembly and budding of enveloped viruses closely resemble the biogenesis of small exosomes, as both depend on cellular membranes. Thus, it is unsurprising that some viruses disrupt MVB formation and exosome production. The ESCRT system plays a key role in enveloped virus production. Studies have shown that many viruses hijack the ESCRT machinery to promote budding, enhancing their replication and spread (Dai J. et al., 2024; Chen et al., 2025). The hijacking of exosome biogenesis by viruses may further enable them to incorporate key components into exosomes, including their own viral nucleic acids, viral proteins, and even complete virions. This phenomenon is particularly evident in the case of HBV.

Exosomes serve as effective carriers of viral DNA and protein components, enabling the transfer of these materials from infected to uninfected cells and thereby promoting HBV dissemination (Wu et al., 2023). HBV-infected cells secrete exosomes that can be isolated from cell culture supernatants, exhibiting characteristic exosomal markers along with HBV-specific components (Wu et al., 2023). These exosome-enriched fractions can be effectively separated from fractions containing free viral particles. Controlled detergent treatment of exosomes leads to the sequential release of intact HBV virions and non-enveloped capsids. Transmission electron microscopy directly visualizes complete HBV virions encapsulated within exosomes (Wu et al., 2023). Additionally, purified extracellular vesicles derived from HBV-infected primary human hepatocytes derived from humanized chimeric mice (PXB) cells contained detectable HBV DNA and exhibited functional transfer competence of viral genetic material to uninfected PXB cells (Sanada et al., 2017). Research has revealed that HBV particles associated with the membranes of late endosomes and large intracellular compartments, suggesting the involvement of the ESCRT mechanism in HBV envelopment (Wang et al., 2020). Disruptions in the ESCRT system hindered HBV budding and release. For example, The knockdown of ESCRT-II not only suppressed the production and release of enveloped HBV virions but also impaired the formation of intracellular nucleocapsids (Stieler and Prange, 2014). Genetic ablation of TSG101 markedly attenuated viral particle secretion, which was corroborated by transmission electron microscopy analysis demonstrating a substantial reduction in HBV virions within MVBs following either TSG101 or NEDD4 depletion (Zheng Y. et al., 2023). HBV employed a sophisticated ERGIC-53-COPII cooperative mechanism for cellular egress, which represented a novel viral export pathway (Zeyen et al., 2020). Evidence has indicated that mutations in critical ESCRT-related genes, such as actin-interacting protein (AIP) 1 or VPS4B, can block the formation of MVBs, subsequently preventing the release of enveloped HBV particles (Lambert et al., 2007). Furthermore, HBx mRNA and protein are packaged within exosomal cargo, shielding them from degradation by host nucleases (Kapoor et al., 2017).

## 4 Exosomes mediate the crosstalk between HBV and immune responses

### 4.1 Exosomes inhibit immune responses in HBV infection

**Figure 2:**
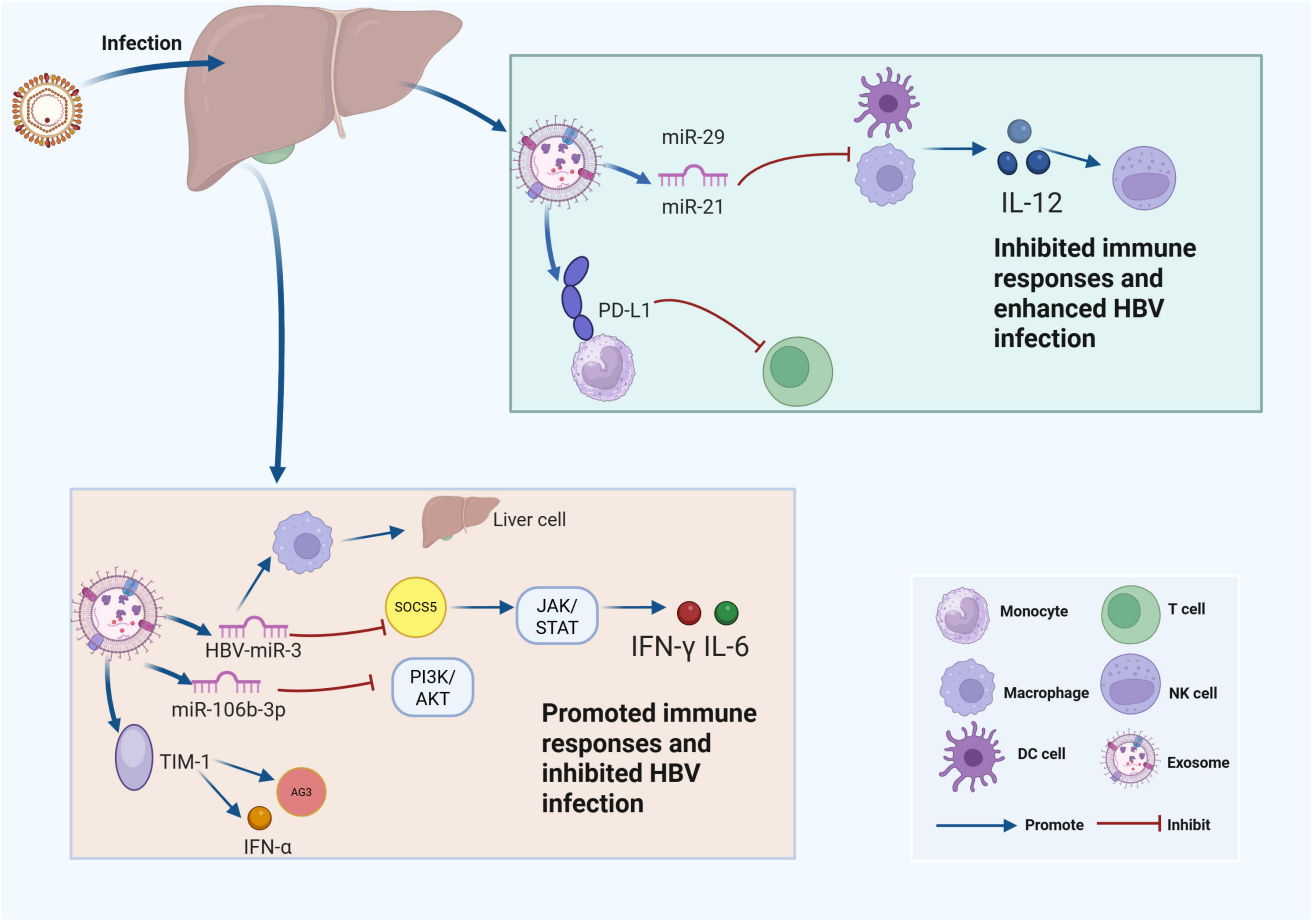
Exosomes influence HBV infection through regulating immune responses. This schematic illustrates the molecular mechanisms by which hepatitis B virus (HBV) modulates host immune responses through microRNAs (miRNAs) and key signaling pathways at the cellular level. Upper right panel: After HBV infection, liver immune cells such as monocytes, macrophages, and dendritic cells (DCs) produce microRNAs including miR-29 and miR-21, which are loaded into EVs. These miRNAs inhibit the expression of interleukin-12 (IL-12), leading to suppressed immune responses and facilitating enhanced HBV infection. In addition, the expression of programmed death-ligand 1 (PD-L1) is promoted by EVs released from HBV-infected cells, further contributing to immune evasion. Lower panel: EVs loaded with HBV-derived microRNAs (e.g., HBV-miR-3) and host microRNAs (such as miR-106b-3p) impact immune pathways within liver cells. HBV-miR-3 upregulates suppressor of cytokine signaling 5 (SOCS5), which suppresses the Janus kinase/signal transducer and activator of transcription (JAK/STAT) signaling pathway, while miR-106b-3p inhibits the phosphoinositide 3-kinase/protein kinase B (PI3K/AKT) pathway. Both mechanisms synergistically enhance the production of interferons, including interferon-gamma (IFN-γ) and interleukin-6 (IL-6), thereby promoting immune responses and inhibiting HBV infection. The interaction between T-cell immunoglobulin and mucin-domain containing-1 (TIM-1) and exosome-associated antigen 3 (Ag3) further stimulates the production of interferon-alpha (IFN-α).

The pathogenesis of chronic hepatitis B (CHB) results from the interaction between HBV and the host immune response (Lok et al., 2016). Consequently, it is unsurprising that HBV-associated exosomes play a critical role in evading the immune system’s defenses. Moreover, exosomes play essential roles in regulating the immune cell function during HBV infection (Liu et al., 2023). Studies have reported that exosomes derived from the serum of CHB patients can facilitate the transmission of HBV to NK cells through the involvement of TGF-β, which leads to impaired NK cell function (Yang Y. et al., 2017). Overexpressed miR-21 and miR-29 in EVs derived from HBV-infected liver cells inhibited the release of IL-12 from dendritic cells and macrophages, leading to impaired NK cell function, inhibited immune response, and progression of liver fibrosis (Kouwaki et al., 2016). Exosomes released from HBV-infected cells have been shown to be endocytosed by monocytes, leading to the upregulation of programmed death-ligand 1 (PD-L1) expression and the simultaneous downregulation of CD69 (Kakizaki et al., 2018). PD-L1, as one of the most critical immune checkpoints, can also be induced by inflammatory cytokines, which ultimately inhibits T cell function (Zhou et al., 2023). CD69 functions as a biomarker of activated immune cells (Yong et al., 2017), which suggests that the depletion and inactivation of T cells in CHB may be attributed to the upregulated PD-L1 expression, which is triggered by HBV-associated exosomes (Kakizaki et al., 2020). In the mouse models of HBV, HBV-derived exosomes significantly suppressed the immune response and exacerbated CHB progression (Kakizaki et al., 2020). These effects were attributed to the impaired clearance of HBV-replicating cells in HBV-infected mice, mediated by HBV-derived EVs (Kakizaki et al., 2020). Moreover, these EVs demonstrated systemic biodistribution, with specific accumulation in multiple organs including the liver, bone marrow (BM), and intestinal tract, suggesting an EV-mediated liver-BM-gut tripartite axis in HBV infection, offering transformative understanding of CHB progression mechanisms (Kakizaki et al., 2020) (**Figure 2**).

### 4.2 Exosomes promote immune responses in HBV infection

Several studies have demonstrated that HBV-associated exosomes can enhance immune responses during HBV infection (Liu et al., 2023). For example, Zhao et al. discovered that hepatitis B virus (HBV) produces a microRNA (HBV-miR-3) that inhibited HBV replication by targeting HBV 3.5-kb transcript, leading to the decreased expression of HBsAg, HBeAg and HBc as well as impaired HBV replication (Yang X. et al., 2017). Further investigations have revealed that HBV-miR-3 activated the JAK/STAT signaling pathway by downregulating suppressor of cytokine signaling 5 (SOCS) in hepatocytes, thereby amplifying the interferon (IFN)-gamma-induced anti-HBV response (Zhao et al., 2020). Moreover, HBV-miR-3 in exosomes promoted M1 macrophage polarization (Zhao et al., 2020). Additionally, exosomes carrying HBV-miR-3 enhanced IL-6 secretion by inhibiting SOCS5-mediated Epidermal growth factor receptor (EGFR) ubiquitination, indicating that HBV-miR-3 activated the innate immune response through multiple pathways to potentially mitigate HBV-induced acute liver cell injury and influencing the HBV infection (Zhao et al., 2020).

In line with this, a study has revealed that liver nonparenchymal cells (LNPCs) treated with IFN-α exhibited increased expression of antiviral proteins, including A3G, MyD88, and PKR, which effectively inhibited HBV infection. Notably, exosomes derived from IFN-α-treated LNPCs were enriched with IFN-α and A3G. These antiviral components within the exosomes could be internalized by hepatocytes, leading to suppressed HBV infection (Li et al., 2013). Moreover, miR-106b-3p has been identified as an interferon-responsive miRNA. Furthermore, interferon could suppress viral replication and antigen expression by delivering miR-106b-3p to HBV-positive hepatocytes via macrophage-derived exosomes, which leading to the suppression of its downstream PI3K/AKT signaling pathway (Chen et al., 2024). Additionally, macrophage-derived exosomes rely on T cell immunoglobulin and mucin receptor 1 (TIM-1) to enter hepatocytes and deliver IFN-α-induced anti-HBV activity. Additionally, lysobisphosphatidic acid (LBPA), an anionic lipid associated with viral endosome penetration, facilitated exosome membrane fusion in late endosomes/multivesicular bodies (LEs/MVBs) and cargo release, providing insights into the transmission mechanisms of macrophage exosomes, highlighting similarities with viral entry pathways (Yao et al., 2018).

## 5 Exosomes as essential biomarkers in HBV-related disease.

HBV infection imposes significant disease burden to public health. Therefore, identifying essential biomarkers to guide treatment decisions and evaluate treatment response are critically important (Wu et al., 2020; Zheng C. et al., 2023). HBV DNA serves as the primary biomarker for viral replication and is the main virological endpoint in clinical trials of nucleos(t)ide analog (NA) therapy (Fung et al., 2022). However, given that current antiviral agents effectively suppress HBV DNA with minimal impact on covalently closed circular DNA (cccDNA), there is a need for additional biomarkers to assess cccDNA transcriptional activity and to evaluate the effects of future antiviral agents (He et al., 2023).

Recently, emerging studies have revealed that EVs have functioned as novel diagnostic and prognostic biomarkers for HBV infection (Wu et al., 2024). Gan et al. reported that HBV-miR-3 in serum-derived exosomes from CHB patients had a positive correlation with HBV DNA, and HBsAg levels. In patients who received NA combined with PEG-interferon (PEG-IFN) treatment, the HBsAg, HBV pregenomic (pgRNA), and HBV-miR-3 exhibited a decrease during the 48 weeks of sequential therapy, indicating that HBV-miR-3 could serve as a supplement to current viral markers (Gan et al., 2022).

The progression to cirrhosis is the most common complication of HBV infection, posing huge health burdens to public health (van Bömmel and Berg, 2013). The onset of cirrhosis is primarily driven by inflammatory activity in the liver, which reflects an immune response to the infection; however, this may not always be indicated by elevated ALT levels, as other contributing factors may also play a role (Guerrieri and Levrero, 2023). Serum EV-associated miR-92a-3p and miR-146a-5p have demonstrated superior diagnostic capabilities compared to the fibrosis index based on four factors (FIB-4), and liver stiffness measurement (LSM) for identifying severe fibrosis patients, thus offering a promising non-invasive alternative (Wang Q. et al., 2021). Serum EV-associated adenosylhomocysteinase (AHCY) expression was also positively correlated with the degree of cirrhosis, achieving over 90% rates of specificity, which exceeded the predictive power of Child–Pugh classification system (Tong et al., 2021).

HBV is the main cause of hepatocellular carcinoma (HCC), which poses a significant public health threat, and early detection remains challenging due to the absence of highly sensitive and specific biomarkers for HBV-related HCC (HBV-HCC) (Papatheodoridi et al., 2022). Todorova et al. employed miRNA sequencing analysis and various bioinformatics methods to identify more than 40 dysregulated exosomal miRNAs in HBV-HCC cells. Among them, miR-483-5p exhibited high specificity and serving as the top dysregulated molecules (Todorova et al., 2023). Moreover, other five miRNAs including miR-182, miR-133a, miR-183, miR-155, and miR-34a positively associated with HBV activity and disease development (Todorova et al., 2023), supporting novel non-invasive biomarkers for the prediction of HBV-HCC. Additionally, exosomal hsa_circ_0028861 was also marked as innovative diagnostic biomarker for HBV-HCC. It exhibited great predictive power for multiple stages and types of HCC including small tumor types, AFP-negative and early-stage phase (Wang Y. et al., 2021).

HBV-related acute-on-chronic liver failure (ACLF) is characterized by elevated short-term mortality rates, highlighting the need for precise prognostic biomarkers for effective early detection and prompt intervention (Wu et al., 2020). By aligning RNA sequencing (RNA-seq), Chen and colleagues revealed that the expression NADPH oxidase 1 (NOX1) and ZSCAN16-AS1 were both upregulated in HBV-ACLF and CHB tissues. Further investigation indicated that both NOX1 and ZSCAN16-AS1 negatively correlated with ALB levels and positively associated with ALT levels, suggesting the promising clinical application of serving as biomarkers for patients with CHB or HBV-ACLF (Chen et al., 2020). In line with this, Xu et al. identified critical small noncoding RNAs (sncRNAs) in HBV-ACLF patients through small RNA-seq approach, which indicating that six sncRNAs including tsRNA-46, tsRNA-20, rsRNA-249, hsa-miR-23b-3p, hsa-miR-223-3p, and hsa-miR-339-5p were uniquely differentially expressed in the plasma exosomes of patients. Moreover, they developed a risk stratification. model called MTR-RNA for HBV-ACLF early detection. The MTR-RNA model exhibited strong predictive power with a 0.787 of C-index, along with more than 70% predictive specificity and sensitivity, which demonstrated that these sncRNAs could function as reliable and feasible biomarkers for HBV-ACLF patients (Xu et al., 2022). Additionally, exosomal lncRNA GAS5 extracted from HBV-ACLF patients exhibited great predictive power of 3-month mortality with achieving AUC of 0.88 (Sun et al., 2023). Exosomal NEAT1 was another biomarker for predicting the 3-month mortality of HBV-ACLF. It exhibited superior predictive power compare to the MELD score system, which attributed to its association with a dysregulated innate immune response that promoted HBV replication, thereby offering a more direct evaluation of the liver microenvironment (Gao et al., 2021). Therefore, exosomes can enhance early diagnosis and offer prognostic insights for HBV-ACLF, assisting in the evaluation of disease progression and informing management adjustments.

Consequently, exosomes have garnered significant attention as potential markers for disease diagnosis. Further exploration of exosomal contents associated with HBV infection is anticipated to facilitate the identification of more promising biomarkers for HBV diagnosis.

## 6 Applications of exosomes in the anti-HBV therapy

Current antiviral therapies for HBV infection are primarily categorized into two classes: interferons (IFNs) and nucleoside analogs (NAs) (Lee et al., 2020). Although IFNs have been extensively used to treat CHB patients, it exhibited limited clinical effectiveness due to the distributions of its antiviral molecular pathways and not fully activated immune responses (Lei et al., 2024). NAs exert their antiviral functions through suppressing the activity of DNA polymerase and impeding the HBV replication progression. However, the clinical effectiveness of NAs remains suboptimal with low proportion of CHB patients achieved HBsAg loss after NAs treatment (Nishikawa et al., 2024). Therefore, there is the urgent need to develop innovative antiviral drugs for impeding HBV replication and achieving favorable clinical efficacy.

Exosomes play essential roles in HBV pathology processes and mediate complex immune responses (Liu et al., 2023). Moreover, they serve as essential biomarkers for the detection and monitoring of HBV-related diseases, suggesting that exosomes hold immense potential for anti-HBV treatment (Hu et al., 2021; Gan et al., 2022). Exosomes could activate immune cells to exert antiviral effects. For example, exosomes containing HBV-miR-3 secreted by HBV-infected hepatocytes can act on macrophages to promote IL-6 secretion and restrict viral replication (Zhao et al., 2020). Additionally, exosomes derived from immune cells could transfer their antiviral capacity to infected hepatocytes. For instance, Exosomes are capable of transferring IFN-α-related miRNAs (hsa-miR-193a-5p, hsa-miR-25-5p, and hsa-miR-574-5p) from macrophages to hepatocytes infected with HBV, which led to significant antiviral activity. Among them, hsa-miR-574-5p bound to the 2750–2757 region of the HBV genome, leading to a reduction in the levels of pgRNA and polymerase mRNA (Wu et al., 2021). Therefore, exosomes could serve as potential agents for antiviral therapy.

Moreover, exosomes could function as the therapeutic targets for HBV infection and HBV-related liver fibrosis. For example, stress-induced tribbles pseudokinase 3 (TRIB3) interacted with sequestosome 1 (SQSTM1) to enhance the secretion of INHBA/Activin A-rich exosomes from hepatocytes, thereby activating hepatic stellate cells (HSCs) and promoting liver fibrosis (Zhang et al., 2020). HBV-infected hepatocytes could also secrete MiR-222 and HBX, which further facilitated the activation of HSCs. Conversely, Kupffer cells produced endogenous miR-690 and transported it via exosomes to hepatocytes, recruited hepatic macrophages (RHMs), and HSCs, directly inhibiting adipogenesis in hepatocytes, suppressing the inflammation in RHMs, and hindering the activation of HSCs (Zhang et al., 2020). Thus, enhancing the secretion of antifibrotic exosomes, inhibiting the release of profibrotic exosomes, or developing therapeutic exosome-based strategies targeting fibrosis may offer promising approaches for delaying the progression of HBV-associated liver fibrosis.

Notably, exosomes could function as carriers by efficiently delivering nucleic acids and proteins that play essential roles in innate immune responses (Buzas, 2023). Exosomes hold great potential to serve as prophylactic vaccines (Kim and Thapa, 2023; Wang et al., 2023). EVs derived from lipopolysaccharide (LPS)-stimulated human monocytic cell line (THP-1) were systematically characterized and demonstrated dual adjuvant properties. Primarily, these exosomes function as natural immunostimulants by potently inducing the secretion of key proinflammatory cytokines including interleukin 1 beta (IL-1β) and tumor necrosis factor alpha (TNF-α), thereby effectively activating host immune responses (Jesus et al., 2018). Furthermore, when co-administered with hepatitis B surface antigen (HBsAg) - either in conventional soluble form or encapsulated in innovative HBsAg-loaded poly-ϵ-caprolactone (PCL)/chitosan nanoparticle delivery systems – the exosomes significantly enhanced vaccine immunogenicity (Jesus et al., 2018). Additionally, Ferrantelli et al. used DNA Vectors to produced engineered exosomes that induced cytotoxic T lymphocyte (CTL) immune responses against HBV-related antigens, which functioned as novel vaccine candidates for anti-HBV treatment (Ferrantelli et al., 2018).

EVs are membrane-delimited nanovesicles derived from biological sources, recognized for their stability, biocompatibility, low immunogenicity, and capacity to traverse biological barriers, which gain recognition as promising vehicles for drug delivery (Kibria et al., 2018). The progress of drug delivery using exosomes is closely associated with advancements in artificial exosome technology and the development of drug-loaded exosomes (Liang et al., 2021). For example, Zeng et al. incorporated vesicular stomatitis virus glycoprotein (VSV-G) onto the EV membrane, which significantly promoted endosomal escape of Clustered Regularly Interspaced Short Palindromic Repeats (CRISPR) -associated protein 9 (Cas9) protein and boosted its gene-editing efficacy in recipient cells. This could effectively reduce viral antigen levels and cccDNA in both HBV replication and infection cell models, indicating that engineered EVs could offer a promising therapeutic approach for the eradication of chronic HBV infection (Zeng et al., 2024).

## 7 Conclusions and perspectives

Exosomes play crucial roles in essential biological processes related to HBV infection. This review provides an overview of research focused on the role of exosomes in the progression of HBV infection, specifically addressing their involvement in HBV replication, synthesis, and release. Drawing on the characteristics and functions of exosomes, we outline and anticipate the potential uses of exosomes in the context of HBV. However, research on exosomes in HBV-related diseases is still in its early stages. Advancing our understanding of the biology of HBV-derived exosomes through further research may lead to the discovery of novel diagnostic and therapeutic targets. While established guidelines are available for the diagnosis and management of HBV infection, additional studies are necessary to develop innovative therapies aimed at suppressing viral replication and transmission while minimizing adverse effects. As highlighted in this review, exosomes play a crucial role in the formation of HBV particles, facilitate viral spread, and modulate the pathogenicity of HBV infection. Therefore, targeting exosome-associated pathways could represent a promising therapeutic approach for HBV infection. Although research into HBV-derived exosomes is still at an early stage, the potential for exosome-based clinical applications is increasingly evident.
